# A complementary role of intracortical inhibition in age-related tactile degradation and its remodelling in humans

**DOI:** 10.1038/srep27388

**Published:** 2016-06-15

**Authors:** Burkhard Pleger, Claudia Wilimzig, Volkmar Nicolas, Tobias Kalisch, Patrick Ragert, Martin Tegenthoff, Hubert R. Dinse

**Affiliations:** 1 Department of Neurology, University Hospital Bergmannsheil, Ruhr-University Bochum, Bochum, Germany; 2 Department of Cognitive Neuroscience, University Hospital Leipzig, Leipzig, Germany; 3 Department of Neurology, Max Planck Institute for Human Cognitive and Brain Sciences, Leipzig, Germany; 4 Institute for Neuroinformatics, Neural Plasticity Lab, Ruhr-University Bochum, Bochum, Germany; 5 Department of Radiology, University Hospital Bergmannsheil, Ruhr-University Bochum, Bochum, Germany

## Abstract

Many attempts are currently underway to restore age-related degraded perception, however, the link between restored perception and remodeled brain function remains elusive. To understand remodeling of age-related cortical reorganization we combined functional magnetic resonance imaging (fMRI) with assessments of tactile acuity, perceptual learning, and computational modeling. We show that aging leads to tactile degradation parallel to enhanced activity in somatosensory cortex. Using a neural field model we reconciled the empirical age-effects by weakening of cortical lateral inhibition. Using perceptual learning, we were able to partially restore tactile acuity, which however was not accompanied by the expected attenuation of cortical activity, but by a further enhancement. The neural field model reproduced these learning effects solely through a weakening of the amplitude of inhibition. These findings suggest that the restoration of age-related degraded tactile acuity on the cortical level is not achieved by re-strengthening lateral inhibition but by further weakening intracortical inhibition.

Aging induces major reorganization and remodeling at all levels of brain structure and function^1^,^2^,^3^,^4^. As a result, sensorimotor and cognitive functions decline progressively. For the sense of touch, numerous studies showed that tactile acuity deteriorates, which is assumed to be due to age-related alterations of the skin and receptor composition as well as of central cortical processing properties^5^,^6^,^7^,^8^. Using electric source localization it was shown that the cortical representations of the fingers are enlarged in somatosensory cortex (SI) of elderly participants parallel to a significant decline of tactile acuity^6^. Numerous lines of evidence converge on the notion that during aging intracortical inhibition is particularly affected, and that much of age-related impairment of sensation and perception may result from this phenomenon^6^,^7^,^9^,^10^,^11^,^12^,^13^. Comparing tactile acuity with intracortical excitability measures obtained in SI showed that excitability in fact increases in individuals of higher age, and that age-related enhancement of cortical excitability correlates with degradation of tactile perception^7^.

Recent research has also shown that age-related changes are not a simple reflection of degenerative processes but a complex mix of plastic adaptive and compensatory mechanisms^3^,^14^,^15^,^16^,^17^, suggesting that neural plasticity is operational at old age. Therefore, many attempts are currently underway to explore the treatability of age-related deterioration^4^,^18^,^19^,^20^,^21^,^22^,^23^,^24^,^25^,^26^. We have recently shown that brief periods of repetitive sensory stimulation are capable of restoring to a substantial amount tactile acuity in elderlies aged 65 to 80 years^21^. While these data demonstrate that age-related decline of sensory capabilities can be significantly ameliorated, the associated neural changes have so far not been addressed.

Combining functional magnetic resonance imaging (fMRI) with assessments of the tactile two-point discrimination threshold, we here first investigated the relationship between age-related alterations in tactile spatial acuity and associated cortical activations in a cohort of healthy elderly individuals aged 51 to 75 years. We then went one step further and studied the nature of restoration of the age-related perceptual decline and of associated cortical reorganization. To this end, we applied a passive tactile stimulation protocol, called coactivation, to the tip of the right index finger over 3 hours to simultaneously stimulate the receptive fields within the skin territory underneath the stimulation device^27^,^28^,^29^,^30^. Tactile coactivation is reliant on the principles of Hebbian learning, according to which precise timing of cortical inputs is a prerequisite to drive plastic changes^31^. In young adults, we had shown that coactivation applied to the right index finger tip led to enhanced neural activation within associated cortical representations parallel to improvements in tactile spatial acuity^29^,^30^. Recent somatosensory evoked potential (SEP) recordings following paired–pulse stimulation showed that these observed cortical changes were accompanied by a reduction of intracortical inhibition^32^. Based on these observations, we hypothesized that coactivation will restore tactile acuity, which will be accompanied by major reorganization in somatosensory cortex most presumably due to reduced intracortical inhibition.

In this view, intracortical inhibition plays a twofold role: there is evidence for an age-related reduction of intracortical inhibition^6^,^7^,^10^,^11^,^12^,^13^, which perceptually is accompanied by a degradation of tactile acuity. On the other hand, the coactivation-induced improvement of acuity is on a phenomenological level of SEP recordings similarly paralleled by reduced inhibition^32^.

To solve this apparent discrepancy, we modeled the underlying processes at the neuronal population level using neural fields with different subgroups of neurons embedded in a topographic cortical representation^33^. In this framework, lateral interaction is a crucial feature, which is assumed to operate through a Mexican-hat-type interaction characterized by recurrent excitation and lateral inhibition^34^,^35^. The activation of neural populations is represented as a one-dimensional cut through the surface of the cortical representation of the index finger tip in primary somatosensory cortex (SI)^33^,^36^. This approach offers the unique possibility to directly link behavior and perceptual performance data to neurophysiological data of cortical processing as obtained through fMRI and SEP recordings^33^. According to our simulations, different aspects of inhibition are responsible for the age-related decline of tactile acuity on the one hand, and of the learning-induced improvement on the other hand. While lateral inhibition is affected by aging, learning targets the amplitude of inhibition. Taking these two complementary roles of intracortical inhibition together, we were able to conceptualize the enhancement of cortical activation as observed empirically during aging and after learning, as well as the opposing effects on perception, where age impairs, but learning improves discrimination.

## Results

### Two-point discrimination thresholds in young and older adults

**Figure 1:**
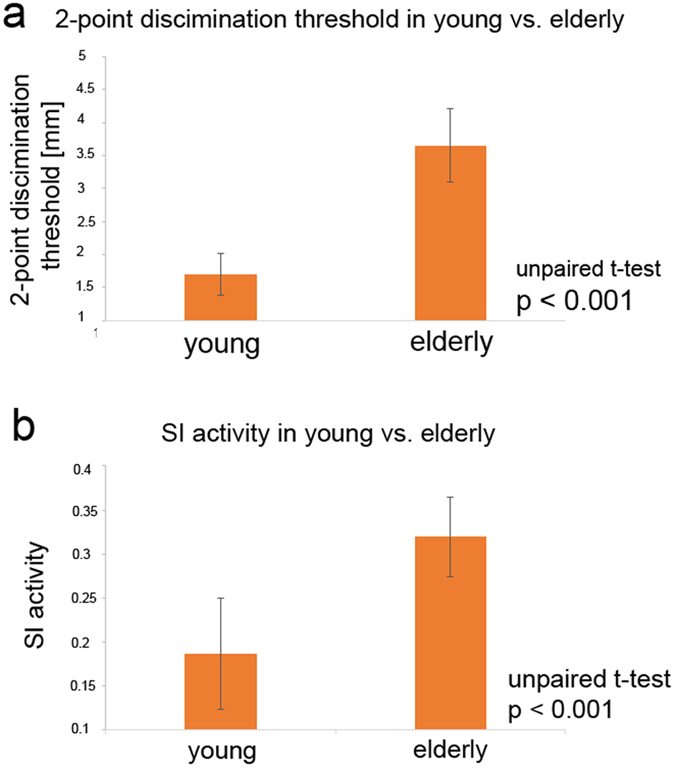
Discrimination thresholds and SI activity in young and elderly participants. Bars indicate the mean and whiskers the standard error. (**a**) The two-sample t-test revealed a significant decline in discrimination acuity in the group of elderly participants. (**b**) SI activity contralateral to the right index finger was significantly higher in elderly as compared to young participants. SI activity was obtained from the SPM one-sample t-test at p = 0.05 (family-wise error corrected) across young and elderly subjects (n = 40) at −30, −34, 62 mm (MNI coordinates), (i.e., same voxels for young and elderly participants), T-value of 5.86 (see Material and methods for further information).

**Figure 2:**
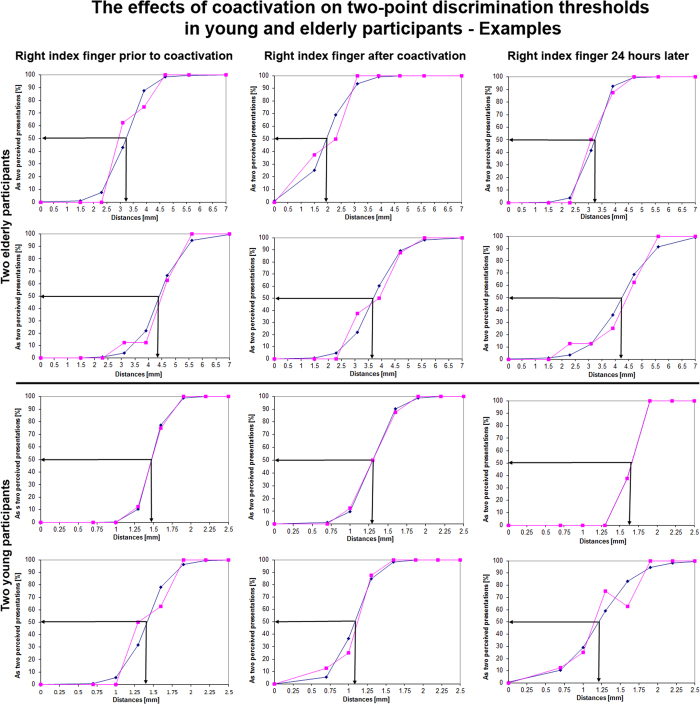
Coactivation effects on two-point discrimination threshold in two representative elderly and young participants. Note that the two-point discrimination thresholds in young participants after coactivation and 24 hours later were published elsewhere^30^ and did not enter any statistical analyses in the present work. These plots are only presented for comparison reasons (i.e., young vs. elderly participants). Shown are the as two perceived presentations in percent (x-axis) for the different tip distances (y-axis). The pink curve indicates participants’ decisions on the different pin distances and the blue curve indicates the fitted binary logistic regression (see Material and methods for further details). The two-point discrimination threshold was determined at the 50% criterion (see black arrows within each plot). Note that 3 hours of tactile coactivation had larger effects on the two-point discrimination thresholds in the elderly as compared to the young participants (see also the Discussion section in the main text for further information).

After each young and elderly participant was familiarized with the two-point discrimination task over three test sessions (64 trials per session), we first assessed baseline two-point discrimination thresholds in 20 healthy young (10 female, age: 25.5+/−3.5 years, mean value+/−standard deviation) and 20 older adults (10 female, age: 64.2+/−6.5 years). Older adults (right index finger (IF): 3.65+/−0.55 mm, left IF: 3.43+/−0.69 mm) showed significantly higher thresholds than young adults (right IF: 1.7+/−0.31 mm, unpaired t-test p < 0.001; left IF: 1.71+/−0.32 mm, p < 0.001, Fig. 1a; see also Fig. 2 for representative psychometric curves in young and elderly participants) indicating age-related degraded tactile spatial discrimination acuity. Suppl. Table 1 lists results from electroneurographic measurements of both median nerves in elderly indicating no degenerative alterations or disturbances of peripheral nerves innervating the index finger.

### The influence of aging on cortical responses

FMRI revealed an enhanced SI activity (thresholded at p = 0.05, family-wise error corrected) within the same SI voxels in the left hemisphere of elderly as compared to young participants (n = 40, p < 0.001, see Fig. 1b). See Suppl. Fig. 1 for the correlation between S1 activity and A. age, B. 2-point discrimination thresholds.

### The influence of coactivation in older adults

**Figure 3:**
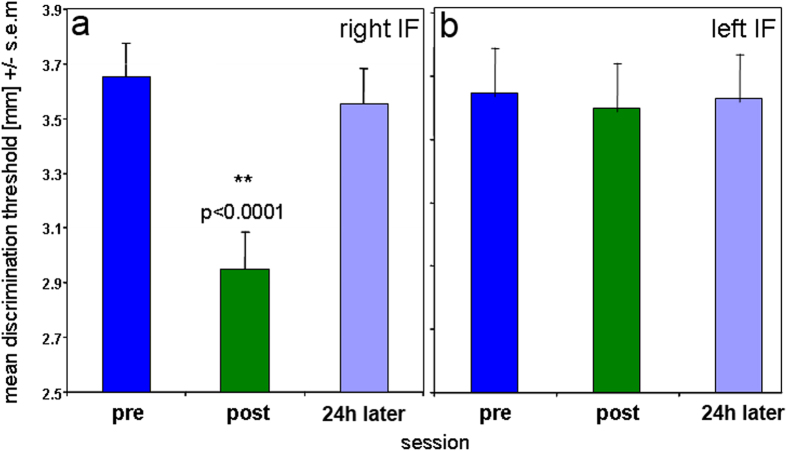
Coactivation influences on discrimination thresholds in elderly. (**a**) Presents the coactivation effects on the tip of the right “coactivated” index finger (IF), and (**b**) the discrimination thresholds of the left “not-coactivated“ IF. Discrimination thresholds obtained for the test finger (right IF) are shown pre- and post coactivation, and 24 h after coactivation. After coactivation was applied to the right IF we found significantly lowered two-point discrimination thresholds (compare pre vs. post) which returned to baseline 24 h later (mean+/−s.e.m.). For the left control IF, thresholds are shown for the same conditions. The general lack of effects for the control finger indicates finger-specificity of the coactivation protocol.

After assessment of baseline two-point discrimination thresholds, tactile coactivation was applied to the tip of the right IF only in the group of the 20 older adults (for coactivation effects in younger adults we refer to^30^). To this end, a small solenoid with a diameter of 8mm was placed on the IF’s tip to simultaneously stimulate (“co-activate”) the skin’s receptive fields underneath the solenoid in a Hebbian fashion^31^. Three hours of coactivation lowered discrimination thresholds from 3.65 mm to 2.95 mm+/−0.6 mm (repeated measures ANOVA (pre, post, 24 hours later): F_(1,38)_ = 37.844; p < 0.001, post-hoc pre-post paired t-test p < 0.001 Bonferroni-corrected, Fig. 3). This equates to a discrimination improvement of 19.2% (normalized to pre session). As an additional control, thresholds of the not-coactivated IF of the left hand remained unchanged (pre: 3.43+/−0.69 mm; post: 3.38 mm+/−0.68 mm, repeated measures ANOVA (pre, post, 24 hours later): F_(1,38)_ = 0.195; p = 0.823, post-hoc pre-post difference t-test p = 0.36, Fig. 3) indicating the local specificity of the coactivation-induced changes. Reassessment of discrimination thresholds twenty-four hours after coactivation revealed two-point discrimination thresholds of the right IF similar to those obtained prior to coactivation (3.55+/−0.57 mm, post-hoc pre-24 hours later paired t-test p = 0.25, Fig. 3; see Fig. 2 for effects of coactivation on psychometric curves in representative young^30^ and elderly participants) indicating reversibility of the coactivation-induced effects. These findings together indicate that the age-related decline in tactile spatial acuity is not irreversible but subject to amelioration through specifically designed sensory stimulation protocols.

### The influence of coactivation on cortical activity in older adults

**Figure 4:**
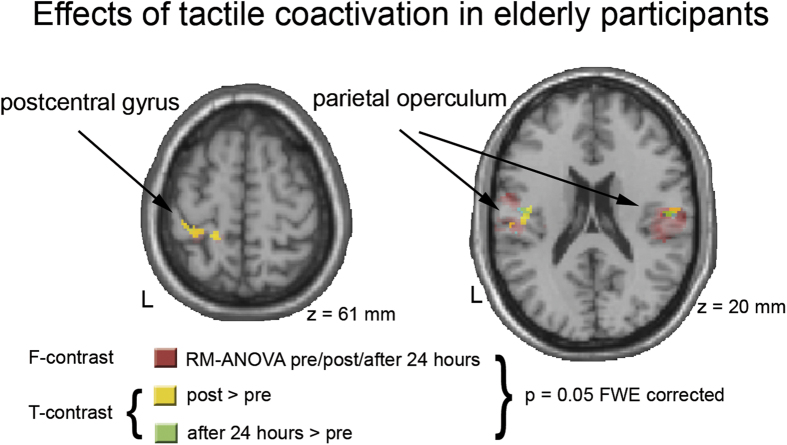
Coactivation effects in the group of elderly participants (n = 20). The ANOVA for repeated measurements (RM-ANOVA or SPM’s one-way ANOVA - within subject) across all three time points (i.e., pre, post, 24 hours after coactivation) of the right (coactivated) index finger revealed significant effects (red clusters) within the left SI (peak voxel: −36, −32, 64 (x, y, z, mm), T = 11.6), as well as in the left SII (peak voxel: −54, −22, 22, T = 53.93) and the right SII (peak voxel: 46, −24, 22, T = 42.72) (restricted to left BA 3b, 1, 2, 4p and bilateral OP1–4). Post-hoc T-contrast comparing the post to the pre session (post > pre, yellow clusters) showed an enhanced SI activation (peak voxel: −32, −36, 62, T = 5.55), as well as an enhanced activation in the left SII (peak voxel: −48, −18, 24, T = 7.73) and the right SII (peak voxel: 54, −12, 18, T = 6.81) due to 3 hours of tactile coactivation. Comparing the data acquired 24 hours after coactvation to the pre session (cyan clusters) revealed no effects in the left SI, but a small activation area within the left SII (peak voxel: −46, −12, 20, T = 5.48) and the right SII (peak voxel: 52, −14, 20, T = 7.21). Together these latter findings suggest complete reversal of the coactivation effects within the SI and an incomplete reversal in the SII 24 hours after tactile coactivation. Note that the effects observed in the SI were always restricted to BA 3b, 1 and 2 and did not spread into the neighboring primary motor cortex (BA 4p). For the left (not coactivated) index finger, we found no significant activity changes across the three time points (i.e., pre, post, 24 hours after coactivation).

After tactile coactivation of the right index fingertip in older adults (n = 20), activity from the contralateral SI and the bilateral secondary somatosensory cortex (SII) increased (Fig. 4). This finding might appear counterintuitive, as aging resulted already in enhanced activation. However, coactivation-related increase in activation from the SI and the SII in older adults is in line with previous observations in young adults^30^.

Comparing the pre coactivation session to the session acquired 24 hours after coactivation, we found no changes in SI activity contralateral to the right ‘coactivated’ IF, but a persistent, albeit lower activation as for the post vs. pre comparison within bilateral SII (Fig. 4). The lack of SI effects suggests that parallel to the reversal seen in the psychophysical data (see ‘*The influence of coactivation in older adults’* and Figs 2 and 3), the enhanced SI activity reversed to the level prior to coactivation within the following 24 hours. The still observable bilateral SII effect after 24 hours suggests an incomplete reversal of the SII activity, which, unlike as for SI, appears incongruent with the complete reversal of the coactivation-induced improvement in tactile acuity. In agreement with our psychophysical findings, we also found no changes in activation by comparing pre and post coactivation session as well as the session acquired 24 hours later of the not-stimulated control IF of the left hand (even if thresholded at p = 0.001, uncorrected), indicating the local specificity of the coactivation effect.

### The neural field model

**Figure 5:**
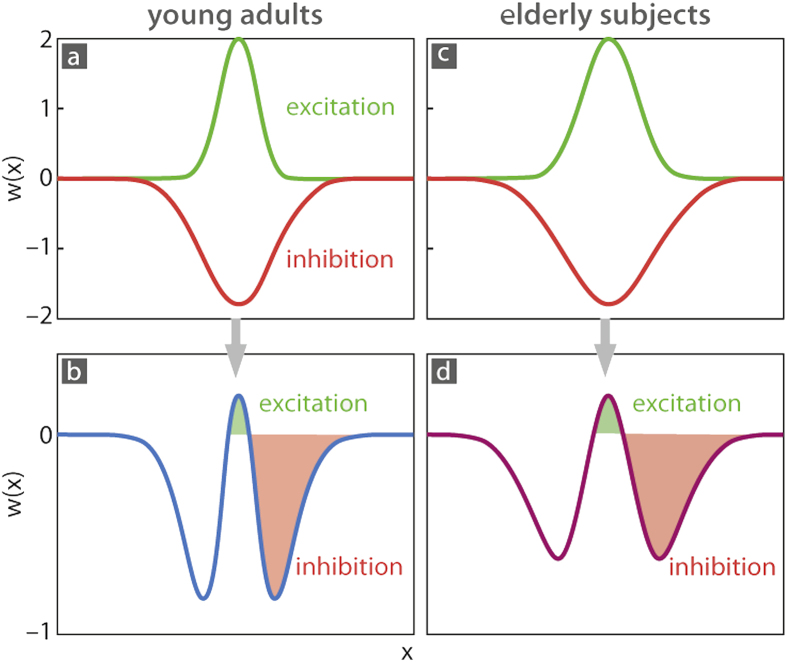
Young and old mean field model. Neural field model of a one-dimensional coronal cut through the surface of the cortical representation of the index finger tip in primary somatosensory cortex. (**a,c**) Interaction arising from each neuron, x, of the modeled dimension (here skin surface) is described by short-range excitatory influences (green) with positive strength (w(x)) and - modulated via the inhibitory layer – broader range inhibition with negative strength w(x) (red). (**b**) The overlay of these two interaction kernels with Gaussian shape, realized by the excitatory and inhibitory layer, results in a Mexican-hat profile of interaction with sharp inhibition (see blue line for an approximation). (**c**) For the model of older adults the widths of the excitatory and inhibitory kernels were assumed to be broader, leading to a broader and more distributed form of interaction. (**d**) The different widths of the interaction kernels result in a different distribution of interaction in the model of older adults characterized by broader excitatory and inhibitory influences (see magenta line for approximation).

To solve the apparent puzzling relation between tactile performance and cortical activation in elderly subjects, we modeled the neural and perceptual data. The model approach introduced 1973 by Wilson and Cowan addresses “cortical” processing assumed to be ubiquitous across all cortical areas independent of modalities and functions. In this sense the model can be seen as universal. The central assumption is that the crucial characteristic of cortical areas is the existence of only two types of neurons, excitatory and inhibitory, though differing in layering and density (for an account of similarities across cortical areas and modalities see^37^). The computational model accounted for different neurons within the neural field interacting through local excitatory –recurrent self-excitation - and longer-range inhibitory connections^34^ (schematically illustrated in Fig. 5; for mathematical details see Materials and methods). As a simplification of underlying neuroanatomy, excitatory and inhibitory neurons were assigned to separate excitatory and inhibitory cortical layers^35^,^38^. While across single neurons, there will be a distribution of different Kernel widths and orientations^39^,^40^,^41^,^42^. At a population level, however, only a single population response is recorded which can be regarded as an average across all contributing single neurons. The MR data we recorded can be seen as an example of such population responses. As the mean field approach also considers population responses, in the model a single kernel is sufficient.

The interaction between these layers (schematically illustrated in Fig. 5; for mathematical details see Materials and methods) was simulated with a Gaussian shaped kernel, which was broader for the inhibitory than for the excitatory layer. This arrangement leads to a Mexican-hat-shaped activation peak with strong winner-takes-all inhibition. As a result, groups of highly activated neurons in a given spatial range cancel out weaker activated neurons, which favors better spatial discrimination especially for intermediate distances. Stimulus input (i.e., single or the two pins of our two-point discrimination test device) and baseline neural activity determined the mutual influence of excitatory and inhibitory layers. Stimulus-dependent activity was simulated by Gaussian distributions and assigned to the single or each of the two activation peaks elicited by the model equivalent of the single or the two pins of the two-point discrimination test device^36^. Thus, simulated cortical activation was shaped not only by the properties and magnitude of the stimulation inputs but also substantially by intracortical interactions.

### Modeling tactile single and two-point stimulation in young adults

**Figure 6:**
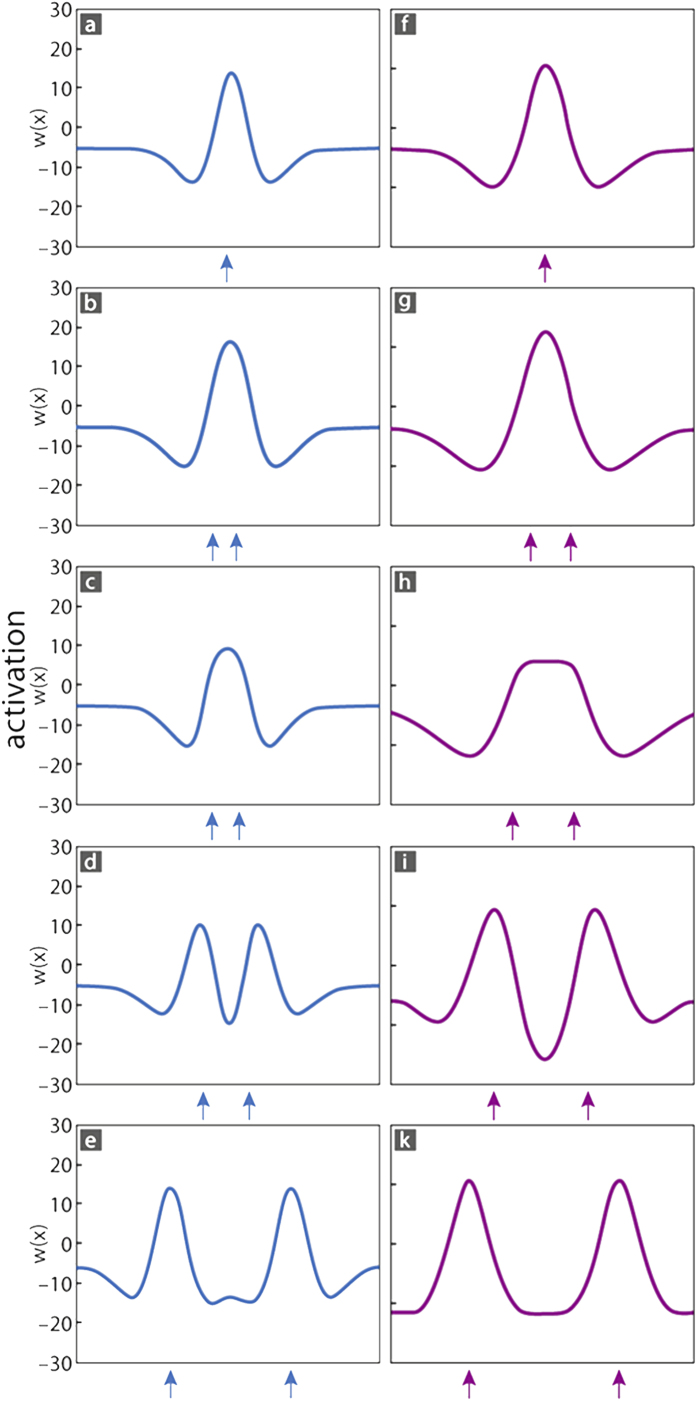
Simulation of age-effects. Stimulation at a single location evokes monomodal distributions of activation with surrounding inhibition. Due to the sharp interaction in the model of young adults the peak of activation is narrow (**a**) but broader and enhanced in the model of older adults (**f**). Inputs from stimulation at two sites (corresponding to the situation during two-point discrimination) at small separation distances fall into the excitatory region, and therefore evoke monomodal distributions (**b,g**). For larger separation distance, the winner-takes-all inhibition becomes strong enough to cancel one of the incoming inputs, leading to a broader, but still monomodal distribution (**c,h**), whose amplitude is reduced due to the strong mutual inhibition. The conditions shown in (**b,g** and in **c,h**) can be regarded as equivalent to perceiving a single pin instead of two. Note that the model behavior is the same for young and older adults, but differs with respect to the equivalence of the separation distances between the two pins of our test device that were larger for older adults (**c,h**). When the separation distances between inputs increased further, the activation distributions become bimodal (**d,i** and **e,k**), which can be regarded as the equivalence for perceiving two separate pins. For intermediate separation distances, the amplitudes of the double-peaks are altered due to the still existing interaction (**d,i**). At large separation distances the evoked distributions remain unaffected from the neighboring distributions (**e,k**). The transition from monomodal to bimodal distributions occur in the model of older adults for larger pin distances (**i,k**).

When the tactile stimulus consisted only of a single pin, our computational model revealed a single peak of Gaussian-shaped activity characterized by a monomodal distribution of activation and lateral suppression due to lateral inhibitory influences (Fig. 6a). When the tactile stimulation consisted of two pins, two peaks of Gaussian-shaped activity emerged. Varying separation distance induced a distant-dependent transition from monomodal to bimodal activation peaks, which can be regarded as reflecting the transition from a single to a two-point percept (compare Fig. 6c with d). Two-point stimulations with small separation distances below threshold evoked mutual excitation, which resulted in a single peak with enhanced amplitude (Fig. 6b,c). With increasing distance between the two stimulation pins, mutual inhibition increased. When the winner-takes-all interaction was sufficiently strong to cancel out one of the inputs, the activation profile remained monomodal resulting in a single pin percept, although two pins were presented. The influence of mutual inhibition was reflected by a reduced amplitude of the monomodal peak (compare Fig. 6b with c). In addition, due to the presented two pins, the resulting peak was broader than for single pin stimulation which occasionally led to a two-point percept.

With increasing separation between the two stimulation pins the distribution of activation became bimodal which can be explained by a decrease in inhibitory interaction between both peaks (Fig. 6d,e). The amplitudes of both peaks were reduced compared to single pin stimulation due to still existent, though weak inhibitory interactions between both peaks. If the distance between the stimulation pins increased further, inhibitory interaction between both peaks decreased and the height of the amplitude became unaffected by the neighboring peak (Fig. 6e).

### Modeling the influence of aging

Previous findings show that the age-related decline in tactile spatial acuity is positively correlated with the degradation of intracortical inhibition^7^, as well as with the enlargement of associated cortical maps^6^. Furthermore, *in-vitro* studies showed that both excitation and inhibition were affected during aging^43^. Based on these findings, and the enhanced activation in SI (see Fig. 1b), as well as the degraded tactile acuity in the group of elderly participants (see Fig. 1a), we simulated age-effects by increasing the width of the kernels representing the mutual interaction of excitatory and inhibitory neural fields. Although the kernel width was the only parameter that was changed, functionally, aging resulted also in a spatial spread of excitation due to the weakened lateral inhibition (Fig. 5).

### Modeling tactile single and two-point stimulation in older adults

The crucial parameter that accounted for aging was the range of intracortical interaction between excitatory and inhibitory components. For young adults the range of interaction was small for both the excitatory and inhibitory components, which resulted in a Mexican-hat function with narrow local excitation and a broader inhibition, but with a pronounced inhibitory surround (Fig. 5). For older adults, the width of both excitation and inhibition was slightly broader. As a result, the inhibitory surround became more distributed and less distinct, which was accompanied by a spread-out of local excitation (Fig. 5). We first simulated a single site stimulation corresponding to situations in which only a single pin is applied to the skin. For both, young and older adults, single site stimulation evoked monomodal distributions of activation (Fig. 6a,f). However, due to the broader range of interaction in the model accounting for older adults, the activity was enhanced and spread-out over the modeled cortical surface (compare Fig. 6 with f), which is equivalent to our imaging data presented in Fig. 1b, and is in line with previously published data about enlarged cortical maps found in older adults^6^. Next, we switched to a condition of stimulating two sites, which corresponds to the situation present during two-point discrimination. When stimulating young or older adults with small separation distances, the intracortical interaction between excitatory and inhibitory components led to a fusion of both inputs, which evoked monomodal distributions of activity (Fig. 6b,c,g,h). This form of activation can be regarded as the equivalent to perceiving a single pin although two pins were applied, but with a distance below the two-point discrimination threshold. In contrast, for larger separation distances, bimodal distributions of activation emerged in young as well as older adults, which can be interpreted as the equivalence to perceiving two separate stimulation pins (Fig. 6d,e,i,k). However, the minimum separation distance between both pins that led to bimodal distributions was larger for older than for young adults, which was due to the fact that in the model of older adults the width of interaction between excitatory and inhibitory components extended over a broader range (compare e.g., Fig. 6c with h). These differences between models for young and older adults predicted the psychophysically observed age-related degradation of discrimination acuity and at the same time accounted for associated enhanced cortical responses. Thus, whether two-point stimulation evoked mono- or bimodal distributions of activity depended on the distance between the pins, which in turn was due to differences in the width of interaction between excitatory and inhibitory components within the modeled cortical topographies.

### Modeling the influence of coactivation

**Figure 7:**
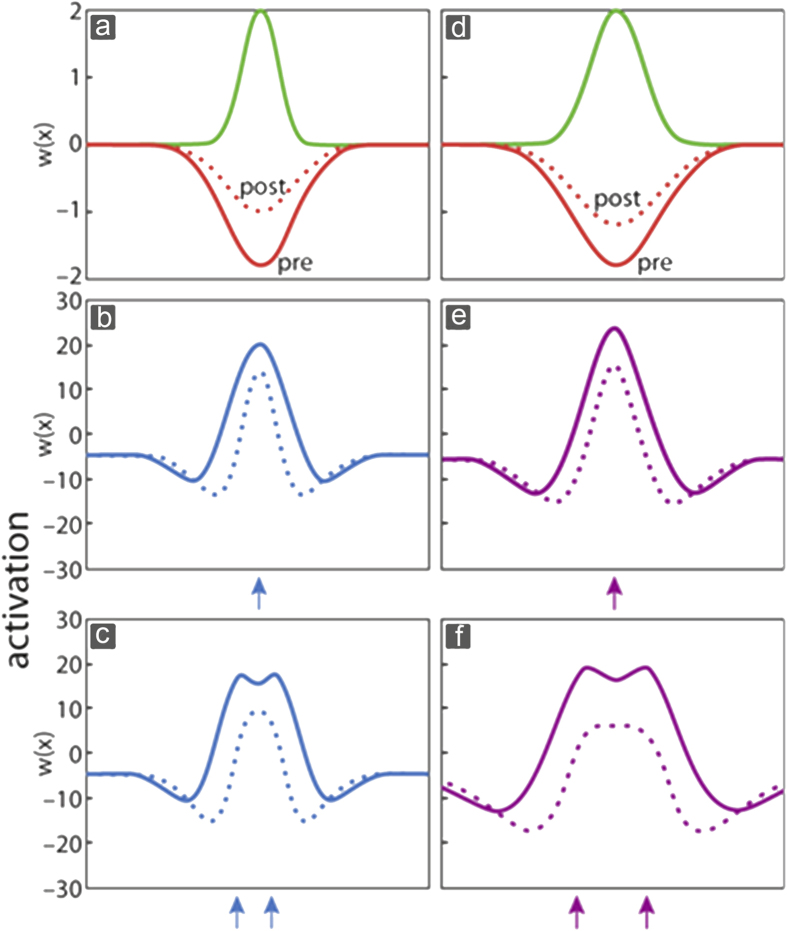
Simulation of coactivation effects. The effect of coactivation is simulated by reducing the amplitude of inhibition depicted by smaller amplitude of the inhibitory kernel both in the model of young (**a**) and older adults (**d**). For stimulation at a single site, this reduction of inhibition leads to both broader and larger peaks of activation in both the model of young (**b**) and older adults (**e**), which corresponds to the enhanced cortical activation seen experimentally (dotted line: pre-condition, solid line: post condition, for experimental data obtained from young adults see ref. 30). At a psychophysical level, the weakening of winner-takes-all mechanisms leads to bimodal activation distributions for separation distances, which under pre-conditions results in monomodal distributions (**c,f**). This situation models the enhanced discrimination abilities observed after coactivation.

In contrast to aging, the effects of tactile coactivation were simulated through a weakening of the inhibitory interaction^33^. Experimental data from human SEP recordings showed reduced intracortical inhibition following coactivation^32^, which was accounted for in both models (for young and older adults, for experimental data obtained from young adults see^30^) by reducing the amplitude of the inhibitory component (Fig. 7a,d). Weaker inhibition allowed for more co-existing excitation. As a result, activation after single site stimulation increased in amplitude (Fig. 7b,e). These changes can be regarded as simulating the enhanced cortical responses observed in both young and older adults after coactivation (see Fig. 4 for older adults, and^30^ for young adults). For conditions of stimulating two sites, the weakening of inhibition allowed the emergence of bimodal distributions of activation. This was true for models of young and older adults for separation distances, which under pre-conditions were characterized by stronger inhibition, and hence resulted in monomodal distributions (Fig. 7c,f). It should be noted that modelling the reduced inhibition in two different ways (weakening of lateral inhibition vs. weakening of the amplitude of inhibition) was the only way to reproduce the empirical data on aging, and on coactivation. In other words, this was not intended, but the outcome of the simulations.

## Discussion

We here used a computational mean field approach to derive a twofold role of cortical inhibitory interaction processes involved in aging and its restoration. Our experimental data demonstrate that aging affects both the layout of cortical representational maps and of tactile spatial acuity. The perceptual decline was not irreparable, but can be partially restored by exposure to repetitive sensory stimulation (i.e., tactile coactivation), which significantly improved tactile acuity. On the other hand, aging resulted in an enhanced cortical activation that paralleled deteriorated perception. Surprisingly, coactivation-induced restoration of acuity, i.e. perceptual improvement, was associated with further enhancement of cortical activation pattern. Accordingly, our data imply two different forms of enhanced cortical responses. The age-related enhancement was accompanied by perceptual decline, but the enhancement following tactile coactivation was paralleled by perceptual improvement, thereby ameliorating the age-related decline.

Degradation of tactile discrimination during aging is a long-known phenomenon^44^,^45^,^46^. Fingertip skin conformance was shown to account for some differences in tactile acuity in young subjects, but not for the decline in spatial acuity with aging^47^. Although there is a loss of mechanoreceptors during aging, the role of peripheral and central age-related changes in mediating reduced tactile acuity are still debated. Several lines of evidence at various levels of neural processing have indicated that inhibition might be reduced in higher age^6^,7,^9^,^10^,^11^,^12^,^13^. For example, enhanced electrical coupling in the hippocampus of aged rats has been reported, which is assumed to contribute to an increase in cellular excitability with age^48^. An ultrastructural study in rats revealed a significant age-related decline in the numerical density of presumptive inhibitory synapses of sensorimotor cortex^9^, demonstrating a deficit in the intrinsic inhibitory circuitry of the aging neocortex. Evidence for a significant degradation of visual orientation and direction selectivity together with enhanced spontaneous activity was described in old macaque monkeys and cats^11^,^12^. The authors suggested that the decreased selectivity and increased excitability of cells in old animals might be attributable to an age-related degeneration of intracortical inhibition. Compatible with these observations, pharmacological experiments in monkeys causally linked age-related degradation of intracortical inhibition to a loss of GABAergic influences^18^. This GABAergic loss could be partially restored by the administration of GABA and muscimol (i.e., GABAa receptor agonist) resulting in improved sensory function, while many sensory cells of old animals displayed responses typical of young cells.

Perceptual improvement can be reliably induced not only by training and practice but also by brief, training-independent sensory learning through repetitive somatosensory stimulation^27^. Tactile coactivation is one of these stimulation protocols closely following the idea of Hebbian learning: Accurately timed neural activity, necessary to drive plastic changes, is evoked by tactile coactivation of the skin thereby linking cellular plasticity mechanisms to human perceptual learning^28^,^30^,^49^. Accordingly, the idea behind coactivation is using the broad knowledge of brain plasticity to design specific sensory stimulation protocols that allow changing brain organization and, thus, perception and behavior through mere expose to sensory peripheral stimulation. Previous imaging and EEG studies demonstrated that after coactivation, which leads to improved acuity, the sensorimotor cortical regions representing the stimulated finger were increased^29^,^30^,^31^,^50^. These findings can be interpreted as a recruitment of processing resources to make processing more efficient. Moreover, intracortical excitability reflecting inhibitory and excitatory processes as studied by using paired-pulse stimulation techniques was enhanced after coactivation, and the amount of enhancement was positively correlated with the individual gain in performance, indicating higher excitability in good learners^32^.

As to underlying biochemical mechanisms of coactivation effects, a single dose of memantine, a selective NMDA receptor blocker, eliminated coactivation-induced learning, both psychophysically and cortically providing strong evidence for the NMDA-R dependence of coactivation-induced learning^28^. Another crucial player is GABA, which plays an important role in the maintenance of the balance of excitation and inhibition. After a single dose of the GABA agonist lorazepam the typically observed improvement of tactile acuity was completely blocked^51^. These studies provide evidence that coactivation induces synaptic plasticity processes are controlled by glutamatergic and GABAergic receptors.

The available data suggest that coactivation drives directly synaptic plasticity processes in the cortical areas representing the stimulated sites. As a result, tactile processing is remodeled, which at a cortical level is expressed as map changes and excitability changes, while perceptually tactile acuity is improved. To explain this effectiveness, a conceptual framework has been suggested, where sensory learning occurs when sensory inputs pass a learning threshold^52^. Under normal conditions, sensory inputs are too weak to pass the learning threshold. Factors that play permissive roles in training-based learning are attention, reward, and motivation thereby amplifying the sensory inputs otherwise below threshold. In case of coactivation and repetitive sensory stimulation approaches, factors such as attention either play no role, or make only a small contribution. Instead, factors that “optimize” sensory inputs are high-frequency or burst-like features as well as heavy schedules of stimulation (i.e. large number of sensory stimuli), which boost inputs that normally are insufficient to drive learning past this learning threshold.

Coactivation in older adults improved tactile acuity from 3.65 mm to 2.95 mm (i.e., Δ = 0.7 mm), whereas young adults in previous assessments^30^ improved only from 1.58 mm to 1.28 mm (i.e, Δ = 0.3 mm). The smaller benefit of young adults together with the higher acuity prior to coactivation suggests a ceiling effect probably due to determined limits of tactile spatial resolution of the skin and cortex at young age. The somatosensory acuity in older adults instead was degraded but the cortex seems to offer even larger plastic capacities as indicated by the coactivation-induced improvement of tactile acuity by 19%, which expressed in years, is equivalent to a gain of 12 years (see Fig. 1a).

In young adults, oral application of lorazepam that enhances GABAa mediated inhibition, completely eliminated the coactivation-induced improvements in tactile spatial acuity^51^. If degradation of intracortical inhibition in older adults depends on a loss of inhibitory GABAergic influences like in old monkeys^18^, the aged cortex could be more susceptible to coactivation resulting in augmented influences on tactile acuity. As described above, in the elderly we found coactivation-induced improvement of 0.7 mm compared to 0.3 mm in young adults^30^, which supports the notion that age-related loss of intracortical inhibition sensitizes the aged cortex to GABA-mediated plastic changes.

To provide an explanation of the process behind both, aging and its restoration through learning processes, we used a neural field model which offers the advantage to link behavior to cortical processing^53^. Crucial parameters in the model are the Mexican-hat-type interaction characterized by recurrent excitation and lateral inhibition. To model the process of aging and learning we solely changed the topographic arrangement of intracortical inhibition, which affected the width of inhibition and excitation. Age-related reduction of inhibition is a well-documented crucial mechanism assumed to explain age-related degradation of sensation and perception^6^,^7^,^9^,^10^,^11^,^12^,^13^. On the other hand, training or stimulation-induced gain of behavioral performance is associated with reduced inhibition^32^,^54^,^55^,^56^,^57^. Implementing these observations in our model, our simulations connected behavior to corresponding cortical map dimensions. As a result, the simulations could capture the experimentally observed age-related degradation of intracortical inhibition^7^, and the enhanced cortical responses in terms of enlarged cortical maps^6^. Similarly, at the perceptual level, the simulations reproduced the age-related decline of discrimination acuity as well as the coactivation-induced enhancement of cortical responses parallel to the perceptual improvement. In order to simulate the aging processes, we increased the width of interacting inhibitory and excitatory kernels, which lead to a lateral spread of excitation due to weakened lateral inhibition. At a functional level, this causes an expansion of cortical representation parallel to impaired discrimination performance. In the simulations, this is visualized by the transition from monomodal to bimodal activation pattern when discriminating larger two-point distances (see Fig. 6c,d,h,i). On the other hand, to simulate discrimination improvement and associated cortical plasticity following coactivation, we reduced the inhibitory amplitude, which also resulted in enhanced cortical activity and enlarged cortical maps. However, due to the reduced inhibitory amplitude, the simulations of the coactivation effects revealed a fundamentally different influence on tactile acuity, namely an improved discrimination performance due to the development of bimodal response peaks indicative of an improved ability to tell two closely neighboring stimuli apart. This mechanism is especially effective for those distances close to discrimination threshold, which after coactivation were perceived as separate and not as a single point as prior to coactivation.

As to the question of scaling, the early developer of these types of models did not comment on the scales of the model in relation to the scale of measureable cortical excitation or inhibition^34^,^35^,^38^. Therefore, our modelling intent was not to quantitatively reproduce the exact spatial scales observed empirically. However, qualitatively the spatial scales used in modeling the aging and the coactivation effects match those observed empirically (Figs 1a,b and 5, 6). Discrimination threshold in the young adult subjects was on average 1.58 mm, and that of the elderly participants 3.65 mm. Correspondingly, in the old model the pin distance, where after coactivation bimodal peaks emerge, is about twofold that of the young model (Fig. 7c,f) thus capturing qualitatively the age-related differences in acuity. As to the size of the coactivation effect, the empirical data showed about 19% improvement. Approximating a threshold in the modeling approach according to Fig. 7b,c,e,f, an improvement after coactivation can be estimated to be in the range of 20 to 30%.

Our simulations of age-related reduced lateral inhibition using a neural field model on the one hand accounted for the enlarged cortical map size together with the lowered tactile acuity prior to coactivation. On the other hand, the reduced lateral inhibition also replicated the higher effectiveness of coactivation in older adults (see Fig. 7, compare differences in simulated response peaks between pre and post coactivation in young adults (b, c) to those of older adults as presented in (e, f). According to our a-priori hypothesis, coactivation can restore some of the age-related degradation of tactile acuity, however, this is accomplished not by a reversal of the age-related enlargement of cortical maps, but instead by a further enlargement. Thus cortical map expansion observed phenomenologically at a level of population activity can be caused by opposing underlying mechanisms, which each have opposing perceptual consequences.

Neural field models have been successfully used to account for the behavior of neural population in motor^58^, visual^59^ as well as in somatosensory cortex^33^,^60^. In the 1970s, it was Wilson and Cowan who introduced this model to explain interactions between inhibitory and excitatory neurons^35^. Later extensions by Amari and Arbib established an effective model for mixed populations of interacting inhibitory and excitatory neurons with typical cortical connections commonly referred to as Mexican hat connectivity^34^,^38^. These extensions made the neural field model specifically effective for modeling sensory map organization since it reproduces the equilibrium of excitatory and inhibitory inputs to pyramidal cells as for instance observed in mouse primary visual cortex^61^. We here used this modelling approach to explore the age-related relationship between tactile acuity and functional organization of cortical maps in human SI. Our simulations showed that it is not the absolute number of neurons that code a stimulus location, but the way they interact through excitation and inhibition.

The simulations of the perceptual findings together with the fMRI results indicate that a single neural field model can link behavior with cortical function to account for aging *and* plastic capacities of the aged somatosensory cortex. In the model, the crucial parameters are the width and amplitude of lateral interaction, which can explain that on the one hand stimulation-induced improvement of tactile acuity is accompanied by enhanced cortical activation, while age-related decline of tactile acuity is phenomenologically accompanied by enhanced cortical activation as well. However, the simulations cannot reveal the underlying mechanisms, by which lateral inhibition is differently affected. There is a large body of evidence about age-related changes of inhibitory processes during aging on many different levels as detailed above such as changes of transmitter release, changes in inhibitory synapses, as well as structural changes. So far it remains unclear whether such changes in concert are sufficient to alter excitation/inhibition in a way suggested by our simulation. Given the complexity of aging, it is conceivable that other mechanisms play a crucial role as well.

A related version of the neural field model used here, the so-called dynamic field model^62^, has recently been employed to simulate changes in spatial cognition abilities from early childhood to adulthood^63^. In this model, the simulation of the cortical development required only modest changes in local excitation to capture age-related changes in behavior. These observations together with our simulations suggest a dynamic, on-going rearrangement of local cortical excitatory and inhibitory processes starting from early childhood to old age and with vast influences on different kinds of behavior. It remains to be clarified in how far other forms of behavior show similar dependencies during development and aging.

Combined, our simulations reproduced complex joint changes of cortical activation and perceptual performance that developed during aging and subsequent learning employed to ameliorate age-related changes. Aging effects on behavior and associated cortical maps can be simply simulated by extending the spread of excitation due to weakened lateral inhibition, whereas tactile coactivation specifically lowered the inhibitory amplitude. As a result we suggest a crucial and complementary role of intracortical inhibition in age-related tactile degradation and its behavioral and cortical restoration.

## Methods

The study was performed in accordance with the 1964 Declaration of Helsinki and approved by the Ethics Committee of the Ruhr-University of Bochum. Subjects gave their written informed consent. We recruited 20 right-handed healthy older adults (10 female, age: 64.2+/−6.5 years (mean value+/−standard deviation), ranging from 51 to 75 years). Twenty right-handed young adults (10 female, age: 25.5+/−3.5 years, ranging from 21 to 32 years) served as controls. To exclude degenerative alterations or disturbances of peripheral nerves innervating the IF, older adults underwent electroneurographic measurements of both median nerves and clinical neurological examination before participation. For none of the subjects we found pathological alterations of the left or the right median nerve (see Supplementary Table 1). In addition, clinical MRI measurements (coronar FLAIR, axial T1-w-SE, axial and sagittal T2-w-TSE, diffusion-weighted EPI sequence and intra- and extracranial MR-angiography) were obtained to exclude structural abnormalities of the brain or its arteries. Any chronic illnesses or central acting medications were exclusion criterions.

### Testing of tactile spatial discrimination

In young and older adults we assessed baseline two-point discrimination thresholds. Seven pairs of pins (for older adults separated by 1.0, 1.4, 1.8, 2.2, 2.6, 3.2, and 4 mm, for young adults by 0.7, 1.0, 1.3, 1.6, 1.9, 2.2, and 2.5 mm) and one single pin were circularly mounted on a rotatable disk. Each single needle had a diameter of 200 microns. The subject’s forearm, hand and fingers were fixed on a plate that, for each presentation, was moved downwards to the rotatable disk with the test IF being placed over a small hole within the plate. The downward-movement was stopped at a fixed position above the pins through which the test IF touched the pins at the same indentations for each presentation. Subjects had to decide immediately after stimulus application if they had the sensation of one or two needles by reporting the percept of a single needle or of doubtful stimulus as “One” but the distinct percept of two stimuli as “Two” According to general practice^28^,^29^,^30^,^39^, each distance was tested 7 times in pseudo-randomized order (56 trials per session) over 15 minutes. The test area on the finger tip was marked with an eudermic pen to ensure that we always applied the discrimination tests as well as the coactivation to the same skin territory.

The percentages of as “two” identified presentations for each distance were fitted by a binary logistic regression (SPSS©, SPSS Inc.). The two-point discrimination threshold was defined as the point on the sigmoid regression curve at which the 50% criterion was reached. Goodness of fit was evaluated by calculating for each subject and for each session linear correlation coefficients between measured and calculated regression values. Across sessions, we found an average (±standard deviation) correlation coefficient (R^2^) of 0.934 ± 0.10 for the group of young adults, and of 0.979 ± 0.035 for the group of older adults.

Threshold differences due to coactivation and 24 h follow-up were tested using the ANOVA and paired t-test, while differences between groups were analyzed using unpaired t-tests. In previous studies we had additionally used d-prime analysis to test for sensitivity changes^30^,^64^. In these studies we found increased d-prime following stimulation. As in the present study false alarm rate was mostly zero, we here abstained from d-prime analysis. To control for unspecific stimulation effect, we previously had used a single-site control stimulation^30^ consisting of only one tiny stimulator (tip diameter 0.5 mm), while frequency and duration of pulses were as described for coactivation. Applying single-site stimulation to the right index finger tip did not change 2-point discrimination thresholds at all, indicating that the coactivation of receptive fields underneath the stimulation device was necessary to induce the observed behavioral and cortical changes^30^. To analyze the consistency and reliability of threshold measurements over sessions, we calculated for each subject Cronbach’s alpha as a marker of test-re-test reliability. In the group of young adults, Cronbach’s alpha was 0.82 ± 0.13 for the right index finger (pre and 24 h follow-up), and an alpha of 0.84 ± 0.12 for the left index finger (pre and 24 h follow-up). In the group of older adults, test-retest reliability was 0.89 ± 0.06 for the right, and 0.92 ± 0.04 for the left index finger.

### FMRI scanning

FMRI scanning was performed with a whole body 1.5 T scanner (Magnetom Symphony, Siemens Medical Systems, Germany) equipped with a high-power gradient-system (30 mT/m/s; SR 125 T/m/s). We used a single-shot SpinEcho-EPI sequence (TR 3000 ms, TE 60 ms, matrix 64 * 64, field of view (FOV) 224 mm, 4-mm slice thickness, 1 mm gap between slices, voxel 3.5 * 3.5 * 4 mm) to assess blood oxygen-level-dependent (BOLD) signals. Twenty-three transaxial slices were adjusted parallel to the AC-PC line which covered the whole brain excluding cerebellum. Cortical responses due to the stimulation of the right and the left IFs were measured in separate sessions. All young subjects (n = 20) were subjected to fMRI once, immediately after the assessment of the two-point discrimination thresholds, whereas the older adults (n = 20) were measured twice, firstly before and secondly after coactivation. In each session we acquired 11 blocks of rest and 10 blocks of activity in alternately order. Each block consisted of 5 scans. For finger stimulation we used a TENS stimulator (Medicommerz, Kirchzarten, Germany) with conventional ring-electrodes (medco) mounted on the tip of the IF. For comparability of these data with previously published fMRI data obtained from young adults^30^, we chose the similar stimulation protocol (pulse duration: 0.1 ms, repetition rate: 3 Hz, stimulation intensity: 2.5 times above sensory threshold). Stimulation electrodes were removed between pre and post coactivation session, but their positions on the fingers were marked to guarantee that the same skin areas were stimulated. Subjects were instructed to keep their eyes closed and to concentrate on the stimulation during the scanning procedure. In all subjects, anatomical images were acquired using an isotropic T1-3dGE (MPRAGE) sequence (TR 1790 ms, TE 388 ms, matrix 256 * 256, FOV 256 mm, 1-mm slice thickness, no gap, voxel size: 1 * 1 * 1 mm) with 160 sagittal orientated slices covering the whole brain.

### FMRI data analyses

For analyzing fMRI data we used the Statistical Parametric Mapping (SPM) software package, version 8 (Wellcome Department of Cognitive Neuroscience, London, UK, http://www.fil.ion.ucl.ac.uk/spm) running under MATLAB R12 (Mathworks Inc., Sherborn, MA). The first 10 images of each fMRI session (115 images) during which the BOLD signal reaches steady state were discarded from further analysis. First, all scans were realigned and unwarped for gradient non-linearity and B0 distortions. Scans were then resliced using Sinc interpolation, followed by the normalization procedure. All volumes were normalized using the standard template of the Montreal Neurological Institute (MNI) (voxel size: 2 mm3). Afterwards, scans were smoothed with a 6-mm (full-width half-maximum) isotropic, three dimensional Gaussian filter. To assess individual pattern of activity we calculated statistical maps using a high-pass cut-off at 256s and a hemodynamic response function (hrf – lowpass filter). On the first (i.e., single-subject) level we modelled the preprocessed MRI images within the general linear model with one regressor representing the blocks of activity. The onsets were convolved with the standard hemodynamic response function as implemented in SPM. The rest blocks entered the baseline the activity blocks were compared to. The T1-GE scans were segmented into gray matter, white matter and cerebrospinal fluid using the “New Segment” algorithm as implemented in the DARTEL (Diffeomorphic Anatomical Registration Through Exponentiated Lie Algebra) toolbox for SPM 8. Next, we applied the “get_totals.m” script bei Ged Ridgway (http://www0.cs.ucl.ac.uk/staff/g.ridgway/vbm/get_totals.m) to the gray matter segments in order to assess the total gray matter volume for each participant. For the topographic assignment of BOLD-signals, we co-registered the mean image formed in a realignment procedure to the T1-GE sequence scan. On the second (group) level we first applied the single-subject contrast images (i.e., activity blocks > baseline) of young and elderly participants (data assessed prior to coactivation) to the one-sample ttest (n = 40). The total gray matter volumes were added as noninteracting covariate to account for age-related gray matter atrophy. Using the anatomy toolbox for SPM by Eickhoff and colleagues^65^ we restricted the analyses to the left Brodman areas (BAs) 3b, 1, and 2 for SI. We extended the SI mask also by the neighboring BA 4p to assess a possible spread of activation into primary motor cortex. To account for bilateral SII activity, we added the 4 areas of the parietal operculum (OP1–4) of each hemisphere. This analyses revealed significant SI activation (p = 0.05, family-wise error (FWE) corrected at the voxel level) with the peak maximum at −30, −34, 62 mm (x, y, z, MNI coordinates) and a T-value of 5.86. We extracted the SI activity from significantly activated clusters. The data was then split into the two age groups (young and elderly participants) and assigned to two-sided and unpaired t-test to assess differences in activity from the same SI voxels between young and elderly participants.

To assess the relationship between BOLD signals, two-point discrimination thresholds and age we computed SPM linear correlation analyses across young and older adults (data assessed prior to coactivation). In the group of older adults (n = 20), changes in SI/SII activation due to coactivation were assessed by the SPM one-way ANOVA (within subject). We defined three regressors of interest (pre coactivation, post coactivation, 24 hours after coactivation) for the right (coactivated) and left (control) index finger. As for the one-sample t-test, we also implemented the total gray matter volumes as a non-interacting covariate. We applied the same SI/SII mask (i.e., left BA 3b, 1, 2, 4p, bilateral OP1–4) as for the one-sample t-test to restrict the analyses to brain regions of interest. To assess the effects across all three sessions for each finger we applied an F-contrast. Post-hoc T-contrasts (post vs. pre, 24 hours later vs. pre) were applied to derive the specific structure of significance. The contrast images were thresholded at p = 0.05 voxel level, FWE corrected.

### Tactile coactivation in older adults

After assessment of the baseline two-point discrimination thresholds, only older adults underwent coactivation procedure as in our previous studies^21^,^28^,^29^,^30^,^49^,^51^ (for coactivation effects in young adults see^30^). Tactile coactivation was applied via a small solenoid with a diameter of 8 mm that was mounted on the tip of the right IF to convey the cutaneous stimuli to the skin. Coactivation-stimuli were presented at different interstimulus-intervals between 100 to 3000 ms in pseudo-randomized order; average stimulation frequency was 1 Hz and the duration of each pulse was 10 ms. Coactivation stimuli were applied at supra-threshold intensities. Duration of coactivation was 3 hours. Pulses were recorded on tape and were played back via portable tape recorders allowing unrestrained mobility of the subjects during coactivation. Subjects were asked to read during the stimulation period and not to attend to the stimulation. Discrimination was always tested within the coactivated skin area.

### Neural field model equations

We used the mathematical approach by Amari and Arbib^34^,^38^ with two separate layers for excitatory (u(x)) and inhibitory neurons (v(x)):


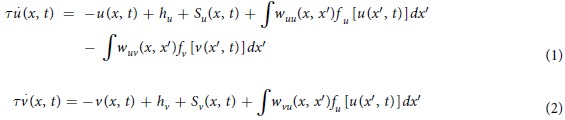


with t representing the time scale of evolution of activity and h_u_ and h_v_ being the resting levels of the respective fields (<0) and with w_uu_, w_uv_ and w_vu_ being the connectivity functions. Only sites that are sufficiently activated participate in the interaction, consistent with the idea that only those neurons which are sufficiently activated can interact with other neurons. This is mathematically expressed by a sigmoidal nonlinearity: m


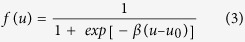


Interaction is excitatory for small two-point distances, inhibitory for medium distances, and zero for larger distances reflecting a Mexican-hat function with winner-takes-all inhibition for medium distances. Stimuli and increased baseline activations for different subgroups are simulated through Gaussian shaped functions:


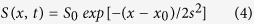


## Additional Information

**How to cite this article**: Pleger, B. *et al*. A complementary role of intracortical inhibition in age-related tactile degradation and its remodelling in humans. *Sci. Rep.* **6**, 27388; doi: 10.1038/srep27388 (2016).

## Supplementary Material

Supplementary Information
