# Supplementary material for: A complementary role of intracortical inhibition in age-related tactile degradation and its remodelling in humans
Source: Sci Rep. 2016 Jun 15;6:27388. doi: 10.1038/srep27388 (PMC4908433; doi:10.1038/srep27388)
Supplement: Supplementary Information [file srep27388-s1.pdf]

Supplementary information for “***A complementary role of intracortical inhibition in age-related tactile degradation and its remodelling in humans***” by

Burkhard Pleger, Claudia Wilimzig, Volkmar Nicolas, Tobias Kalisch, Patrick Ragert, Martin Tegenthoff, & Hubert R. Dinse

A.

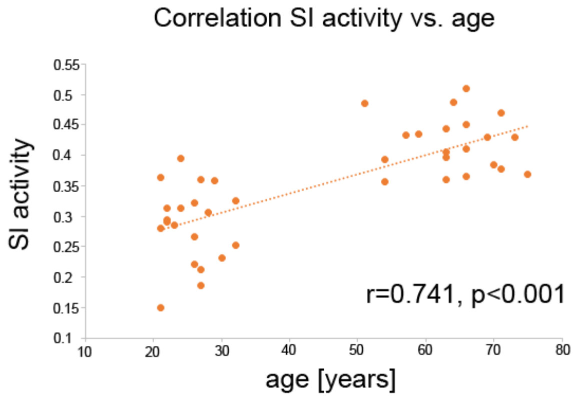

B.

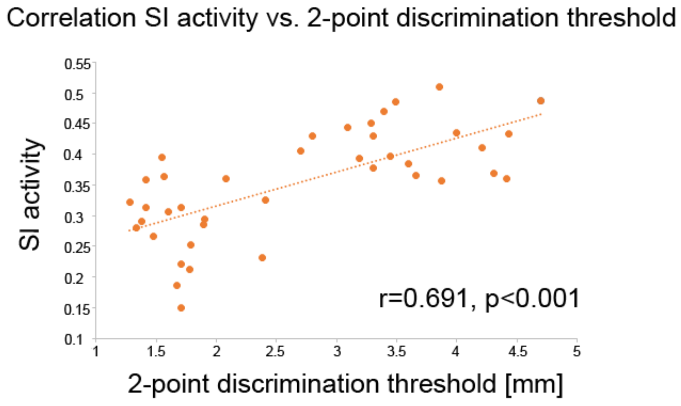

Supplementary Figure 1.

Correlation between S1 activity, age and 2-point discrimination thresholds **A.** Correlation between fMRI activity, obtained from the S1 region of interest, and age [years] across young and older adults (n=40) revealed a positive correlation (r=0.741, p<0.001). **B.** Correlation between S1 activity and two-point discrimination thresholds [mm] revealed also a positive correlation (r=0.691, p<0.001). These finding suggest that the more advanced the age and the worse the 2-point discrimination thresholds, the higher the S1 activity was.

Sets of model parameters:

The purpose of the model here is to present processing principles emerging during aging; and in the context of learning processes, without explicitly addressing the scaling. Even the developer of these types of models [1-3] did not comment on the scales of the model in relation to the scale of measureable cortical excitation or inhibition.

τ (time scale) of excitatory layer:	-3 to -4
τ (time scale) of inhibitory layer:	-3 to -4
Resting level (h) of excitatory layer:	-50
Resting level (h) of inhibitory layer:	-10
β (for sigmoidal nonlinearity) for both the excitatory and the inhibitory layer:	1.5
Threshold for interaction:	0
Amplitude of the stimulus (modeled as a Gaussian shaped function):	7.5 – 10
Effective width of excitation for young subjects:	5 – 10
Effective width of excitation for old subjects:	15
Effective width of inhibition for young subjects:	15 – 20
Effective width of inhibition for old subjects:	25 – 30

Please note that the width of the excitation and inhibition are codependent and their respective influence on the overall cortical interaction depend on their mathematical interaction. All parameters other that the width of interaction were kept constant between young and old subjects.

1. Amari, S. Dynamics of pattern formation in lateral-inhibition type neural fields. Biol. Cybern. 27, 77-87 (1977).
2. Wilson, H.R., & Cowan, J.D. A mathematical theory of the functional dynamics of cortical and thalamic nervous tissue. Kybernetik 13, 55-80 (1973).
3. Arbib, M.A. & Amari, S. Sensori-motor transformations in the brain (with a critique of the tensor theory of cerebellum). J. Theor. Biol. 112, 123-155 (1985).

Supplementary Table 1. Electroneurographic measurements of both median nerves in elderly indicate no degenerative alterations or disturbances of peripheral nerves innervating the index finger.

**Electroneurography**		**right median nerve**		**left median nerve**	
		Mean	*SD*	*mean*	*SD*
**Sensible**	conduction velocity (m/s)	52	2.5	53.5	2.6
	amplitude (µV)	12	2.8	12.9	4.7
**Motor**	distal motor latency (m/s)	1.7	0.24	1.7	0.16
	conduction velocity (m/s)	56	3.6	55.1	3.7
	amplitude (µV)	8.9	2.7	10.1	3.3
